# Parallel-Coupled Microstrip-Lines-Based Miniaturized Balanced Bandpass Filters with Flexible Differential-Fed I/O Ports

**DOI:** 10.3390/mi16111238

**Published:** 2025-10-30

**Authors:** Chuan Shao, Guijie Liu, Rong Cai, Rongchang Jiang, Xinnai Zhang, Kai Xu

**Affiliations:** 1 School of Information Engineering, Jiangsu College of Engineering and Technology, Nantong 226000, China; guijieliu@jcet.edu.cn (G.L.); caironghopeness@hotmail.com (R.C.); rongchjiang@jcet.edu.cn (R.J.); zhangxinnai@163.com (X.Z.); 2 Nantong Key Laboratory of Artificial Intelligence New Quality Technology, Jiangsu College of Engineering and Technology, Nantong 226000, China; 3 Nantong Key Laboratory of Advanced Microwave Technology, Nantong University, Nantong 226019, China; xukaihopeness@hotmail.com

**Keywords:** flexible input/output, balanced filter, miniaturized size, common mode rejection, wideband response

## Abstract

In this paper, a miniaturized balanced bandpass filter with flexible input/output (I/O) functionality is initially designed based on parallel-coupled microstrip lines. Unlike conventional balanced bandpass filters, the proposed filter features two distinct I/O configurations. In these two states, the I/O ports of the developed balanced filter are symmetrically arranged in either horizontal or vertical directions. Moreover, the developed balanced filter demonstrates excellent differential-mode and common-mode suppression in both states. To further enhance the common-mode suppression without compromising the differential-mode performance, an asymmetrical quarter-wavelength open-circuited stub is introduced in the middle of the filter when the I/O ports are vertically symmetric. The inclusion of this stub significantly broadens the common-mode suppression bandwidth. More importantly, the developed balanced filters achieve highly compact sizes, which is essential for integration into modern compact RF front-end modules. To verify the feasibility of the proposed design concept, two prototypes are designed and fabricated, whose simulated and measured results are in good agreement.

## 1. Introduction

In contemporary wireless communication systems, balanced bandpass filters have become essential functional units, indispensable in applications that extend from fifth generation mobile terminals to internet of things sensor nodes [1,2,3,4,5]. By selectively passing the desired differential-mode signals while simultaneously attenuating unwanted common-mode noise, these filters safeguard signal integrity and minimize electromagnetic interference. Their ability to deliver high common-mode rejection across wide bandwidths makes them a cornerstone of high-performance RF front-end modules, where stringent spectral efficiency, low insertion loss, and compact integration are simultaneously demanded.

Over the past few years, a variety of architectures and methodologies have been proposed for balanced bandpass filters [6]. The primary research orientations of the proposed designs are systematically directed towards the substantial enhancement of stopband rejection for balanced bandpass filters [7,8,9,10], the rigorous sharpening of passband selectivity [11,12,13,14,15,16], the progressive elevation of common-mode suppression by means of the synergistic integration of diversified transmission-line topologies [17,18], and so forth.

To accomplish harmonic suppression, a wide suppression bandwidth was achieved in [7,8] through the deliberate adoption of specialized coupling schemes. In [9], the desired suppression was likewise realized by exploiting stepped impedance resonators whose higher-order modes are purposely suppressed. Furthermore, in [10], perturbations were judiciously introduced within the substrate-integrated waveguide so that spurious harmonics were effectively shifted to higher frequencies, thereby yielding an extended spurious-free response.

In order to markedly enhance the passband selectivity for balanced filters, hybrid electromagnetic-coupling modes were deliberately employed in [11]. Subsequently, in [12,13,14], multiple transmission zeros were strategically generated through the incorporation of bandstop sections, thereby securing a sharply steep passband transition. Moreover, in [15,16], supplementary transmission zeros were created via cross-coupling paths, which, in turn, enabled an aggressively sharpened selectivity.

A substantially elevated common-mode rejection level together with an appreciably broadened suppression bandwidth is customarily pursued through the deliberate co-deployment of heterogeneous transmission-line topologies [17,18]. This strategy is primarily motivated by the intrinsic common-mode suppression capability exhibited by certain guiding structures—slotline being a representative example [18]. Nevertheless, the introduction of slotline inevitably necessitates the disruption of the ground plane; consequently, balanced bandpass filters synthesized via this approach are almost invariably implemented on multilayer substrates.

For traditional differential-fed (balanced) bandpass filters mentioned above, the assignment of differential-fed ports to the input and output is typically invariant, whereby the plane of structural symmetry is inherently established. When this fixed port assignment is subsequently altered, the differential- and common-mode responses are observed to be entirely inconsistent with those previously obtained. In this paper, miniaturized balanced filters based on parallel coupled microstrip lines are proposed. The symmetric plane of the developed balanced filter can be extended in both the horizontal or vertical directions, and good differential-mode response and common-mode suppression can be achieved in both cases, which have not been realized in previous balanced filters.

## 2. Working Principles of the Developed Balanced Bandpass Filters

**Figure 1:**
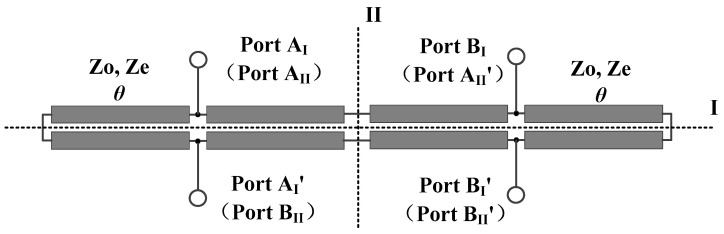
Transmission line model of the proposed balanced bandpass filter.

The transmission line model of the proposed balanced wideband bandpass filter with flexible input/output (I/O) functionality is depicted in Figure 1. As illustrated in the figure, the developed balanced bandpass filter is constituted by four pairs of parallel-coupled microstrip lines with electrical length of *θ* and two sets of balanced ports. It is noteworthy that, owing to its precisely mirrored geometry, this filter exhibits two distinct symmetry planes, subsequently designated as Plane I and Plane II.

When Plane I is taken as the plane of symmetry, the two pairs of differential-fed ports are Port A_I_ and Port A_I_′, and Port B_I_ and Port B_I_′ respectively. When Plane II is taken as the plane of symmetry, the two pairs of differential-fed ports are Port A_II_ and Port A_II_′, and Port B_II_ and Port B_II_′ respectively. Thus, an analysis of the balanced wideband bandpass filter is conducted with Plane I or Plane II as the plane of symmetry.

### 2.1. Analysis with Plane I as the Symmetry Plane

**Figure 2:**
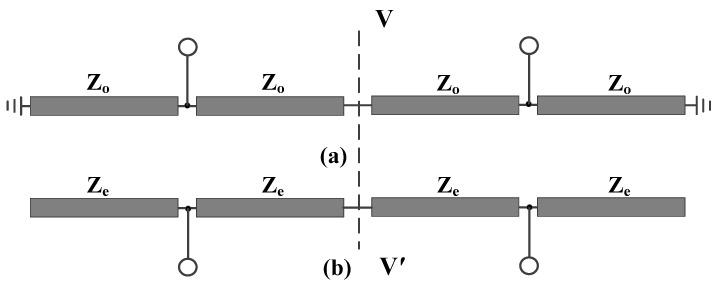
Differential- and common-mode equivalent circuits of the developed balanced bandpass filter with Plane I as the symmetry plane, (**a**) differential mode, (**b**) common mode.

**Figure 3:**
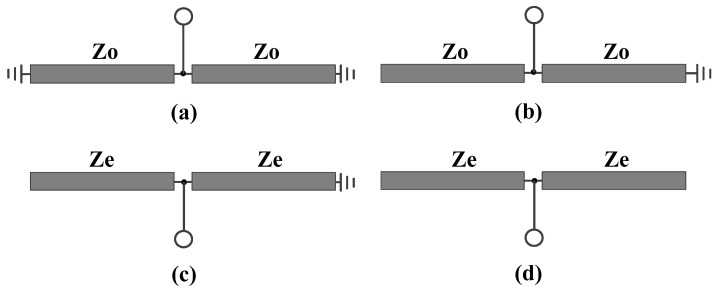
Equivalent circuits of the differential-mode equivalent circuit and the common-mode equivalent circuit, (**a**) odd mode for the differential-mode equivalent circuit, (**b**) even mode for the differential-mode equivalent circuit, (**c**) odd mode for the common-mode equivalent circuit, (**d**) even mode for the common-mode equivalent circuit.

When Plane I is taken as the plane of symmetry, the proposed balanced bandpass filter is subjected to odd-even mode analysis. The resulting differential and common mode equivalent circuits, as shown in Figure 2, are both symmetric with respect to the symmetry plane VV′. Subsequently, an additional odd-even-mode analysis is performed on the aforementioned differential- and common-mode equivalent circuits. As a result, the odd-even-mode equivalent circuits of the differential-mode equivalent circuit and the common-mode equivalent circuit are, respectively, illustrated from Figure 3a,d.

A. Differential-Mode Analysis

For the odd-mode equivalent circuit of the differential mode equivalent circuit, its input admittance is given by:

(1) $$Y_{\mathrm{inDMo}}=\frac{2}{jZ_{o}\tan\theta }$$

When *Y*_inDMo_ is set to zero, the resonance frequency of the equivalent circuit is determined to be π/2, where the differential-mode response exhibits a transmission pole located precisely at the center frequency *f*_0_ for the differential-mode response.

As for the even-mode equivalent circuit corresponding to the differential-mode equivalent circuit, the input admittance is represented by:

(2) $$Y_{\mathrm{inDMe}}=\frac{1}{jZ_{o}\tan\theta }-\frac{1}{jZ_{o}\cot\theta }$$

Given the relationship between *S*_dd11_ and the odd-mode and even-mode input admittances (*Y*_inDMe_ and *Y*_inDMo_) for the differential mode [19], the following relationship can be obtained:

(3) $$S_{\mathrm{dd}11}=\frac{Y_{0}^{2}-Y_{\mathrm{inDMe}}Y_{\mathrm{inDMo}}}{\left(Y_{0}+Y_{\mathrm{inDMe}}\right)\left(Y_{0}+Y_{\mathrm{inDMo}}\right)}$$

Setting *S*_dd11_ = 0 allows the identification of two transmission poles (*θ*^I^_p1_, *θ*^I^_p2_) for the differential mode, which can be derived as follows:

(4) $$\theta_{p1}^{I}=\mathrm{acrtan}\sqrt{\frac{2Z_{o}^{2}}{2Z_{o}^{2}-Z_{0}^{2}}}$$

(5) $$\theta_{p2}^{I}=\pi -\mathrm{acrtan}\sqrt{\frac{2Z_{o}^{2}}{2Z_{o}^{2}-Z_{0}^{2}}}$$

where Z_0_ is the characteristic impedance of the input and output ports.

With respect to Equation (4), when the impedance ratio Z_0_/Zₒ is set to *K*, the expression can subsequently be transformed into the following form:

(6) $$\theta_{p1}^{I}=\mathrm{acrtan}\sqrt{\frac{2}{2-K^{2}}}$$

As indicated by Equations (4)–(6), these two transmission poles are symmetrically distributed around the center frequency, and their locations are determined solely by the value of *K*. Specifically, a larger *K* results in a wider separation, whereas a smaller *K* yields a closer spacing. Subsequently, when the condition Z_0_ = Zₒ is satisfied, the positions of the two transmission zeros can be explicitly derived from the expression given below:

(7) $$\theta_{p1}^{I}=54.7{}^{\circ}$$

(8) $$\theta_{p2}^{I}=125.3{}^{\circ}$$

**Figure 4:**
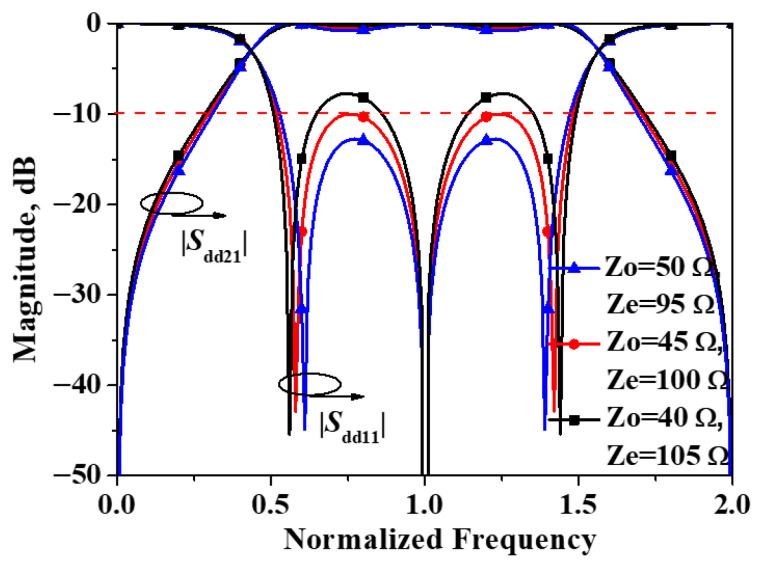
Simulated |*S*_dd21_| and |*S*_dd11_| of the developed balanced filter exhibiting mirror symmetry with respect to Plane I.

Simulated |*S*_dd21_| and |*S*_dd11_| of the developed balanced filter exhibiting mirror symmetry with respect to Plane I versus differential combination of *Z*_o_ and *Z*_e_ are plotted in Figure 4. As can be observed from the figure, the simulated results are in close agreement with the previously computed structure, and the positions of these three transmission poles likewise coincide with the theoretical predictions. Additionally, it is worth noting that, given the symmetry of the two transmission poles about the center frequency, the differential-mode response of the filter exhibits equal ripple performance, that is, the Chebyshev transfer function [20].

B. Common-Mode Analysis

As for the even-mode equivalent circuit corresponding to the common-mode equivalent circuit in Figure 3d, the input impedance is given as:

(9) $$Z_{\mathrm{inCMe}}=\frac{2}{jZ_{e}\tan\theta }$$

When *Z*_inCMe_ is set to zero, the transmission zero of the equivalent circuit is located precisely at π/2, i.e., a transmission zero in the common-mode response appears at the center frequency *f*_0_ of the differential-mode response.

**Figure 5:**
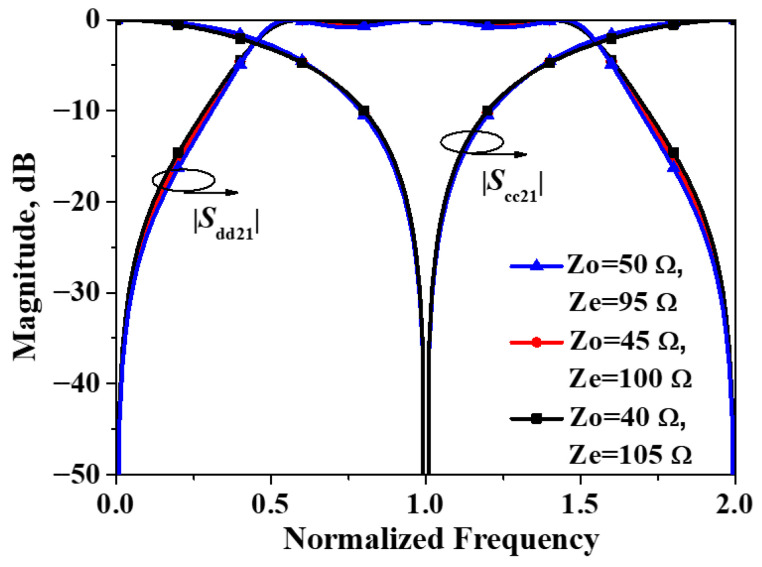
Simulated |*S*_dd21_| and |*S*_cc21_| of the developed balanced filter exhibiting mirror symmetry with respect to Plane I.

Simulated |*S*_dd21_| and |*S*_cc21_| of the developed balanced filter exhibiting mirror symmetry with respect to Plane I versus differential combination of *Z*_o_ and *Z*_e_ are plotted in Figure 5. As illustrated in the figure, the simulated results are consistent with the preceding theoretical predictions. A transmission zero in the common-mode response appears precisely at the center frequency for the differential-mode response, and the common-mode response remains essentially invariant for all combinations of *Z*_0_ and *Z*ₑ considered.

### 2.2. Analysis with Plane II as the Symmetry Plane

**Figure 6:**
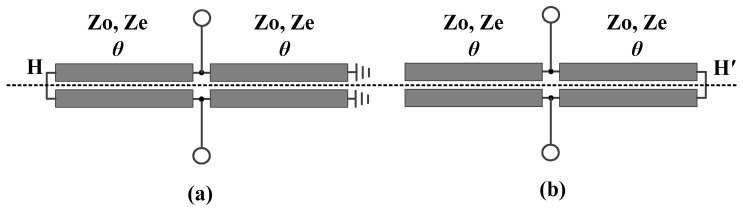
Differential- and common-mode equivalent circuits of the developed balanced bandpass filter with Plane II as the symmetry plane, (**a**) differential mode, (**b**) common mode.

**Figure 7:**
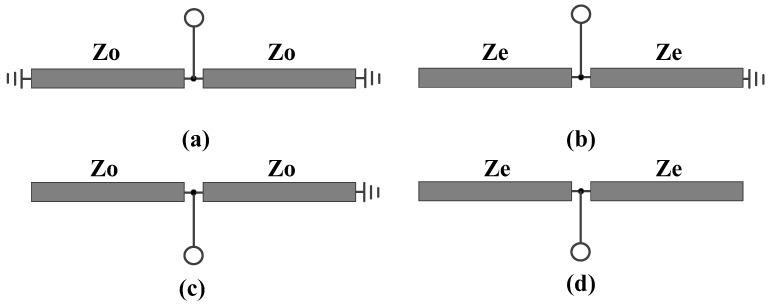
Equivalent circuits of the differential-mode equivalent circuit and the common- mode equivalent circuit, (**a**) odd mode for the differential-mode equivalent circuit, (**b**) even mode for the differential-mode equivalent circuit, (**c**) odd mode for the common-mode equivalent circuit, (**d**) even mode for the common-mode equivalent circuit.

When Plane II is taken as the plane of symmetry, the developed differential-fed bandpass filter is also subjected to odd-even mode analysis. As illustrated in Figure 6, the derived differential- and common-mode equivalent circuits are each symmetric about the HH′ mirror plane. Subsequently, an additional odd-even-mode analysis is performed on the aforementioned differential and common mode equivalent circuits. Thereafter, an additional odd-even-mode analysis is performed on the differential- and common-mode equivalent circuits, and the resulting odd- and even-mode equivalents are, respectively, illustrated in Figure 7a,b for the differential-mode circuit and Figure 7c,d for the common-mode circuit.

A. Differential-Mode Analysis

Following the analytical methodology presented in Section 2.1, the input admittances of the odd- and even-mode equivalent circuits for the differential-mode equivalent circuit shown in Figure 7 can be determined as follow:

(10) $$Y_{\mathrm{inDMo}}=\frac{2}{jZ_{o}\tan\theta }$$

(11) $$Y_{\mathrm{inDMe}}=\frac{1}{jZ_{e}\tan\theta }-\frac{1}{jZ_{e}\cot\theta }$$

Consequently, three transmission poles can be derived using the same analytical approach as detailed in Section 2.1, as follows:

(12) $$\theta_{p1}^{\mathrm{II}}=\pi /2$$

(13) $$\theta_{p2}^{\mathrm{II}}=\arctan\left(\sqrt{\frac{2Z_{0}^{2}}{2Z_{0}^{2}-Z_{o}Z_{e}}}\right)$$

(14) $$\theta_{p3}^{\mathrm{II}}=\pi -\theta_{p2}^{\mathrm{II}}$$

With respect to Equation (13), when the ratio $Z_{0}^{2}$/Z_o_Z_e_ is set to *P*, the expression can subsequently be transformed into the following form:

(15) $$\theta_{p2}^{\mathrm{II}}=\arctan\left(\sqrt{\frac{2P^{2}}{2P^{2}-1}}\right)$$

As indicated by Equations (13)–(15), these two transmission poles are symmetrically distributed around the center frequency for the differential-mode, and their locations are determined solely by the value of *P*. Subsequently, when the condition $Z_{0}^{2}$ = *Z*_o_*Z*_e_ is satisfied, the positions of the two transmission zeros can be explicitly derived from the expression given below:

(16) $$\theta_{p1}^{\mathrm{II}}=54.7{}^{\circ}$$

(17) $$\theta_{p2}^{\mathrm{II}}=125.3{}^{\circ}$$

It is evident that these two transmission poles coincide exactly with the positions of the transmission poles in the differential-mode response as discussed in Section 2.1.

**Figure 8:**
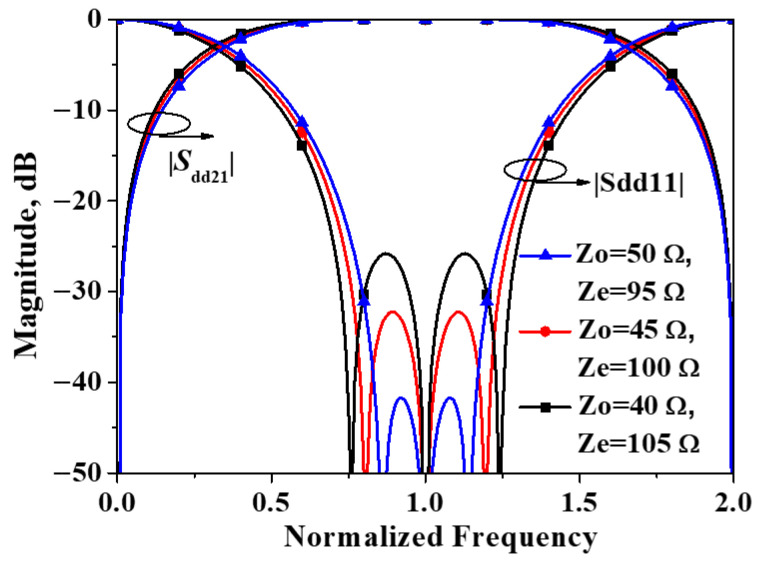
Simulated |*S*_dd21_| and |*S*_dd11_| of the developed balanced filter exhibiting mirror symmetry with respect to Plane II.

The simulation results for the magnitudes of |*S*_dd21_| and |*S*_dd11_| of the designed balanced filter, which display mirror symmetry relative to Plane II across various differential impedance combinations of *Z*_o_ and *Z*_e_, are depicted in Figure 8. As can be observed from the figure, the outcomes align with the earlier theoretical projections.

B. Common-Mode Analysis

Similarly, by referring to the analytical approach outlined in Section 2.1, the input admittances for the odd and even modes of the common-mode equivalent circuit shown in Figure 7 can be obtained, which are as follows:

(18) $$Y_{\mathrm{inCMo}}=\frac{1}{jZ_{o}\tan\theta }-\frac{1}{jZ_{o}\cot\theta }$$

(19) $$Y_{\mathrm{inCMe}}=-\frac{2}{jZ_{e}\cot\theta }$$

When *Z*_inCMe_ is set to be the reciprocal of *Y*_inCMe_, which equals zero, a transmission zero in the common-mode response of the balanced filter is identified to be situated at π/2, which corresponds to the center frequency *f*_0_ for the differential-mode response.

Given the relationship between *S*_cc21_ and the odd-mode and even-mode input admittances (*Y*_inCMo_ and *Y*_inCMe_) for the common-mode equivalent circuit [19], the following relationship can be obtained:

(20) $$S_{\mathrm{cc}21}=\frac{Y_{0}^{2}(Y_{\mathrm{inCMo}}-Y_{\mathrm{inCMe}})}{\left(Y_{0}+Y_{\mathrm{inCMe}}\right)\left(Y_{0}+Y_{\mathrm{inCMo}}\right)}$$

setting *S*_cc21_ = 0 allows the identification of another two transmission zeros for common-mode response, which can be derived as follows:

(21) $$\theta_{z1}^{\mathrm{II}}=\mathrm{acrtan}\sqrt{\frac{1}{1-2Z_{o}/Z_{e}}}$$

(22) $$\theta_{z2}^{\mathrm{II}}=\pi -\theta_{z1}^{\mathrm{II}}$$

**Figure 9:**
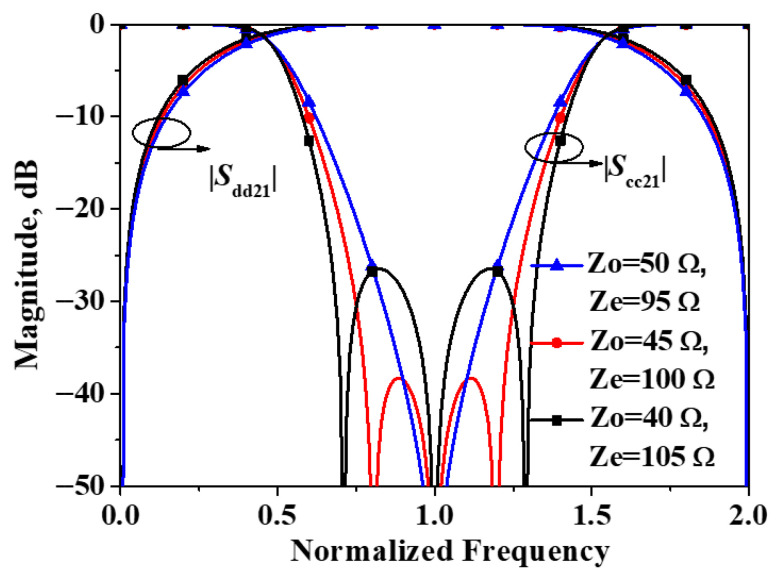
Simulated |*S*_dd21_| and |*S*_cc21_| of the developed balanced filter exhibiting mirror symmetry with respect to Plane II.

As indicated by Equations (21) and (22), these two transmission zeros are symmetrically distributed around the center frequency for the differential-mode, and their locations are determined solely by *Z*_o_/*Z*_e_. Additionally, the existence of these two transmission zeros is contingent upon the condition that 2*Z*_e_ < *Z*_o_. When 2*Z*_e_ > *Z*_o_, these zeros cease to exist. This phenomenon is also evident from the results shown in Figure 9. Specifically, when *Z*_e_ = 50 Ω and *Z*_o_ = 95 Ω, the common-mode transmission coefficient exhibits only one transmission zero.

### 2.3. Comparison Between Two Different Feed Combinations

**Figure 10:**
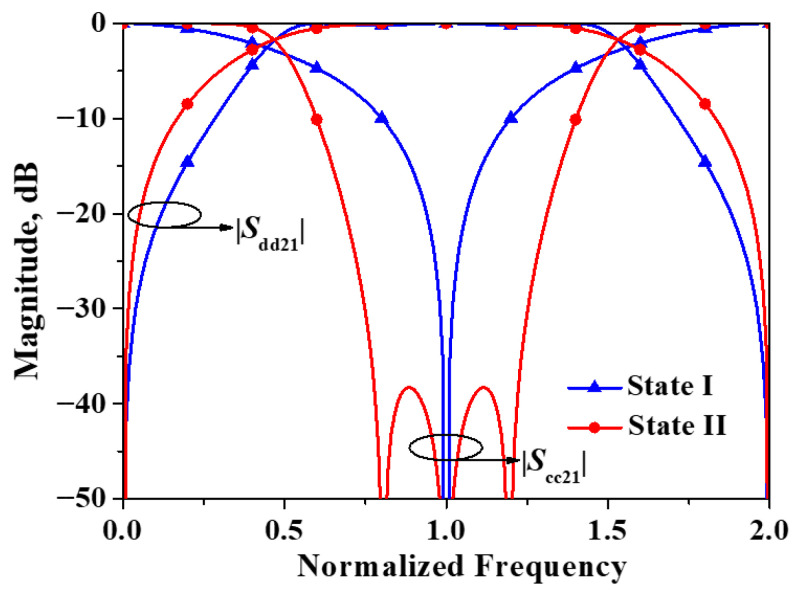
Simulated |*S*_dd21_| and |*S*_cc21_| of the developed balanced filter with flexible differential-fed ports (*Z*_o_ = 45 Ω, *Z*_e_ = 100 Ω).

In order to compare the differential and common-mode responses corresponding to the two different feed combinations, the differential and common-mode transmission coefficients of the two cases are simulated, as shown in Figure 10. For the sake of convenience, the balanced filter with Plane I as the symmetry plane is referred to as State I, and the balanced filter with Plane II as the symmetry plane is referred to as State II.

As can be seen from Figure 10, the two states exhibit nearly identical bandwidths in terms of the differential-mode response. However, compared with State II, State I demonstrates superior differential-mode passband selectivity. Regarding the common-mode response, both states achieve significant suppression at the center frequency of the differential-mode response. Nevertheless, State II exhibits a much wider common-mode suppression bandwidth than State I. In summary, both states display satisfactory differential and common-mode responses, with each state possessing its own distinct advantages.

### 2.4. Modified Structure of the Developed Balanced Bandpass Filter

**Figure 11:**
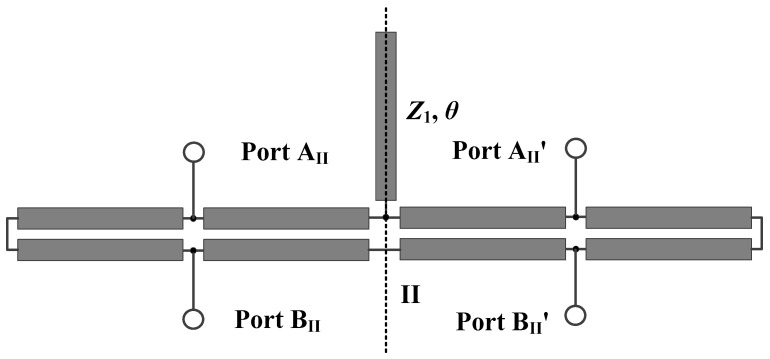
Transmission line model of the modified structure of developed balanced filter with flexible differential-fed ports.

In order to further enhance the common-mode suppression bandwidth of State II, improvements are made to the balanced filter described previously. The structure of the improved filter is shown in Figure 11. A segment of open-circuit stub with a length of *θ* and characteristic impedance of *Z*_1_ is introduced in the middle of the filter.

**Figure 12:**
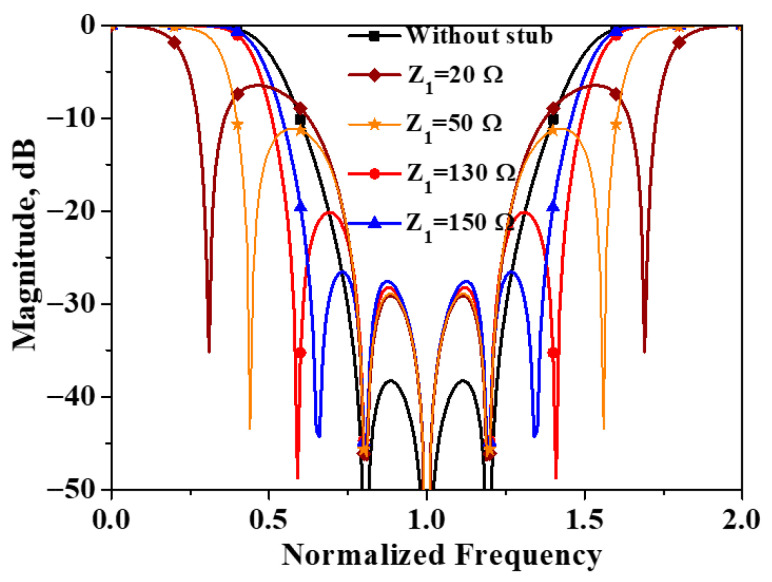
Simulated |*S*_cc21_| of the modified structure of developed balanced filter with flexible differential-fed ports.

The simulated |*S*_cc21_| of the modified structure versus available characteristic impedance [21] of the loaded stub is plotted in Figure 12. As can be observed from the figure, the introduction of the open-circuit stub results in the creation of two additional transmission zeros in the common-mode transmission coefficient, without affecting the three previously existing transmission zeros. Additionally, this significantly extends the common-mode suppression bandwidth of the filter. It is worth noting that as the value of Z_1_ decreases, the distance between the two newly added transmission zeros gradually increases. That is to say, the smaller the impedance of the open-circuit stub, the farther apart the two additional transmission zeros are, which in turn leads to a wider common-mode suppression bandwidth. The specific working principle has been elaborated in detail in the literature [22]. However, this also results in a reduced level of common-mode suppression. Therefore, with a common-mode rejection level of 20 dB as the criterion, the characteristic impedance of the open-circuited stub is selected to be 150 Ω.

## 3. Results and Discussions

**Figure 13:**
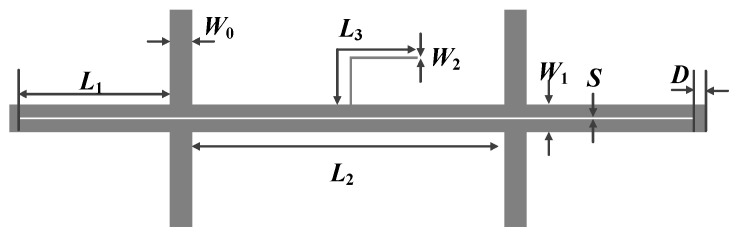
Layout of the developed balanced bandpass filter (*W*_0_ = 1.95 mm, *L*_1_ = 14.9 mm, *L*_2_ = 30.4 mm, *L*_3_ = 15.7 mm, *W*_1_ = 1.8 mm, *W*_2_ = 0.1 mm, *S* = 0.2 mm, *D* = 0.3 mm).

**Figure 14:**
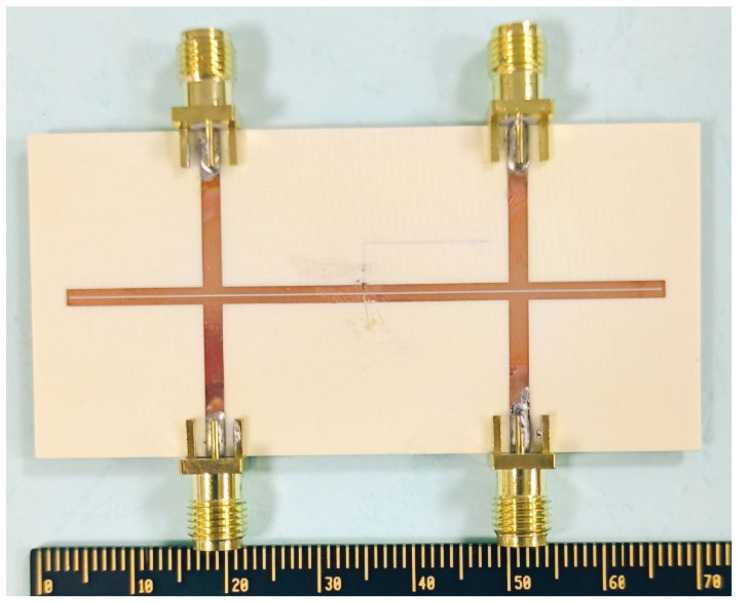
Photograph of the developed balanced bandpass filter with flexible input/output configuration.

On the basis of the aforementioned theoretical derivation and analysis, the odd-mode and even-mode impedances are selected to be 45 Ω and 100 Ω, respectively, in order to ensure satisfactory performance of the balanced filter under the two states. Moreover, the center frequency of the differential mode of the filter is determined to be 3 GHz. Additionally, the substrate employed is Rogers 4003c (a loss tangent of 0.0027, a dielectric constant of 3.55, and a thickness of 0.813 mm). The layout and photograph of the proposed balanced bandpass filter with flexible I/O functionality are given in Figure 13 and Figure 14, respectively.

**Figure 15:**
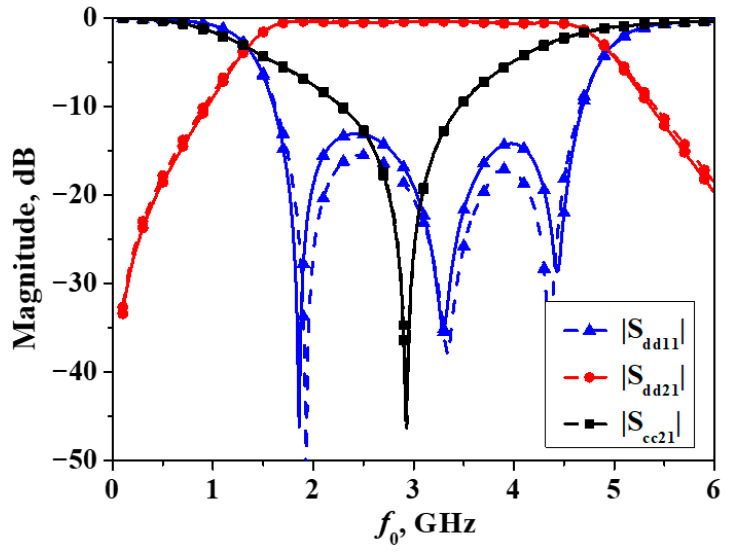
Simulated and measured results of the developed balanced filter exhibiting mirror symmetry with respect to Plane I (Simulation: dash line, measurement: solid line).

The simulation and measurement of the developed balanced bandpass filter are, respectively, accomplished using the full-wave simulator Ansys HFSS and the Keysight 5230C four-port vector network analyzer. When this filter is symmetric along the horizontal direction, the measured and simulated results are depicted as shown in Figure 15. It can be observed from the figure that the center frequency of the differential-mode response of the filter is 3 GHz, with a 3-dB passband bandwidth of 3.2 GHz, corresponding to a relative bandwidth of 106%. Within the 3-dB differential-mode bandwidth, common-mode signals are suppressed to varying degrees, with the maximum level of suppression exceeding 45 dB.

**Figure 16:**
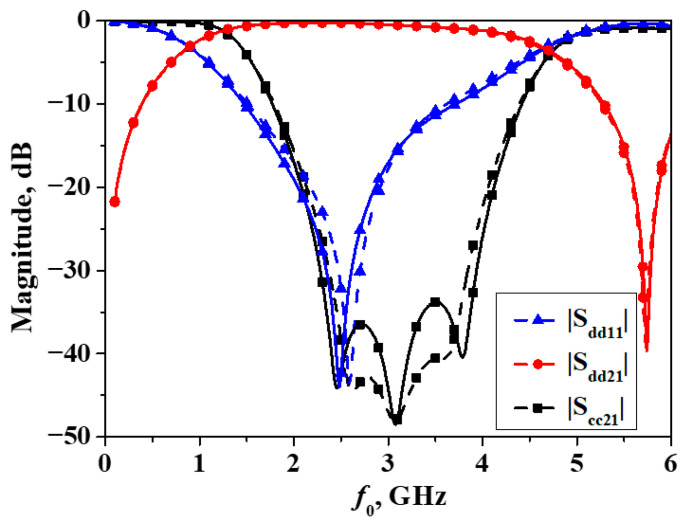
Simulated and measured results of the developed balanced filter exhibiting mirror symmetry with respect to Plane II (Simulation: dash line, measurement: solid line).

When the filter is symmetric along the vertical direction, the simulation and measurement results of the differential-mode and common-mode responses are illustrated as shown in Figure 16. As can be seen from the figure, the center frequency of the differential-mode response is also centered at 3 GHz, and the 3-dB passband bandwidth of the differential mode is also approximately 3.2 GHz, which is essentially consistent with that of the filter when it is symmetric along the horizontal direction. Within the frequency range of 2.1~4.06 GHz, the common-mode signals are suppressed by more than 20 dB, with the highest level of suppression being greater than 45 dB.

**Figure 17:**
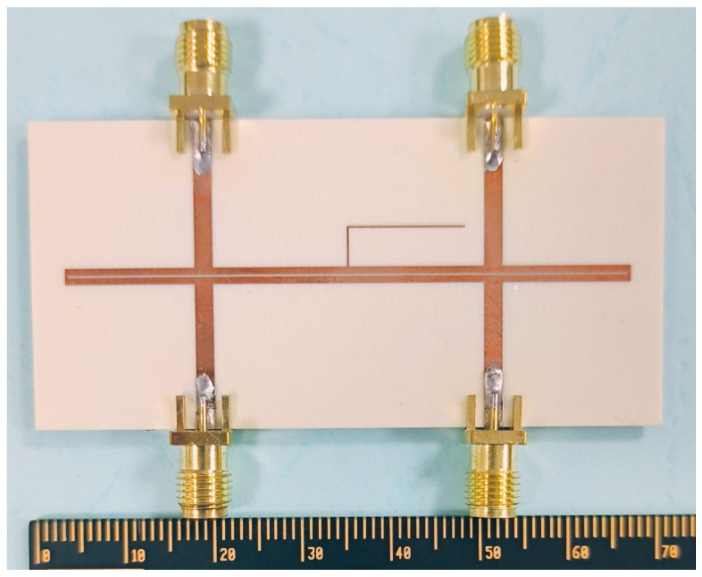
Photograph of the modified balanced bandpass filter.

**Figure 18:**
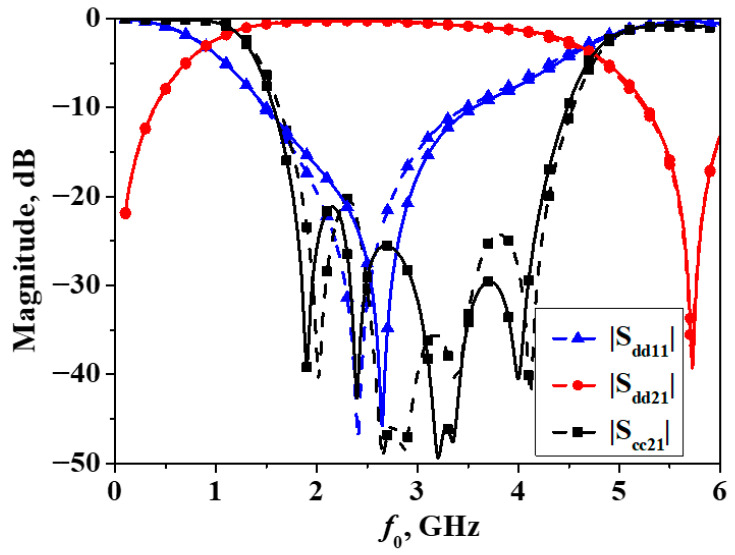
Simulated and measured results of the modified balanced bandpass filter (Simulation: dash line, measurement: solid line).

In order to further enhance the common-mode rejection level for State II, an asymmetric quarter-wavelength open-circuited stub (corresponding to the center frequency) has been loaded in the middle of the developed balanced filter with flexible differential-fed I/O ports, the photograph of which is shown in Figure 17 and the simulated and measured results are plotted in Figure 18. It can be observed that the addition of the open-circuited stub introduces two additional transmission zeros in the common-mode transmission coefficient, located on either side of the original three transmission zeros. In addition, compared with the filter without the open-circuited stub, the differential-mode response remains essentially unchanged. For the common-mode rejection, the 20-dB common-mode rejection bandwidth has been increased from 2.1~4.06 GHz to 1.76~4.25 GHz. In comparison, the 20-dB common-mode rejection bandwidth has been enhanced by approximately 28%. Meanwhile, the size of the filter has not increased significantly.

To further demonstrate the performance of the proposed balanced bandpass filters, comparisons with state-of-the-art designs are tabulated and listed in Table 1. As shown in Table 1, in terms of common-mode rejection level and bandwidth, the balanced filters proposed in this paper are comparable to the previously reported single-layer filters. Moreover, the two balanced filters proposed in this paper both feature a compact size, which significantly reduces the space occupied by the circuit. Most importantly, a balanced filter with flexible differential-fed I/O ports has been realized in this study, which represents a novel functionality not previously achieved in existing structures. In addition, both states of this filter exhibit satisfactory differential-mode and common-mode responses, allowing for the selection of different states according to specific requirements.

## 4. Conclusions

In this study, a miniaturized broadband balanced bandpass filter has been initially proposed, which is distinguished by its flexible input/output functionality. When different input/output ports are selected, the filter exhibits similar differential-mode responses, and the corresponding common-mode rejection is found to be acceptable. Such functionality has not been achieved in previous balanced filter designs. Additionally, when the input/output ports are symmetric along the vertical direction, an asymmetrical quarter-wavelength open-circuited stub is introduced in the middle of the filter in order to further enhance the level of common-mode suppression. The incorporation of this stub not only increases the 20-dB common-mode suppression bandwidth but also extends it from 65% to 83%. During this process, the size of the filter remains essentially unchanged, and the differential-mode response is unaffected.

## Figures and Tables

**Table 1:** Performance summary of the proposed balanced filters and state-of-the-art designs.

Reference		*f*_0_, GHz/ *FBW*	Insertion Loss, dB	Layer	CM Suppression Level and Bandwidth	Flexible I/O Ports	Size *λ*_g_ × *λ*_g_
[7]		2.4/16%	1	Single	10 dB (DC~10 GHz)	NO	0.42 × 0.05
[8]		2.4/11.3%	1.2	Multi	20 dB (DC~6 GHz)	NO	0.53 × 0.26
[9]		0.92/8.4%	2	Single	40 dB (DC~4 GHz)	NO	NA
[10]		12/6%	1.1	Multi	20 dB (10~18 GHz)	NO	1.4 × 1.4
[11]		2.47/6.4%	1.74	Dual	40 dB (DC~6 GHz)	NO	0.49 × 0.24
[12]		4.08/34.6%	1.36	Dual	40 dB (1~7 GHz)	NO	NA
[13]		3.7/92%	1.23	Dual	20 dB (2~5 GHz)	NO	NA
[14]		2.43/6%	0.8	Single	25 dB (1~3.5 GHz)	NO	0.54 × 0.33
[15]		2.45/16.3%	1.3	Dual	30 dB (DC~10 GHz)	NO	0.62 × 0.57
[16]		2.66/13.8%	2.9	Single	38 dB (1~5 GHz)	NO	0.49 × 0.34
[17]		6.6/116%	NA	Dual	30 dB (1~12 GHz)	NO	NA
[18]		3.5/5%	2.56	Single	30 dB (3~4 GHz)	NO	0.89 × 0.83
This works	State I	3/106%	0.4	Single	5 dB (1~4.5 GHz)	YES	1 × 0.03
	State II				20 dB (2.1~4.06 GHz)		
	Modified structure				20 dB (1.76~4.25 GHz)	NO	1 × 0.1

λ_g_ is the guided wavelength at the center frequency.

## Data Availability

The original contributions presented in this study are included in the article. Further inquiries can be directed to the corresponding author.
